# Light Scattering Measurements on Solutions of Some Quaternary Ammonium Salts

**DOI:** 10.6028/jres.068A.035

**Published:** 1964-08-01

**Authors:** Stanley P. Wasik, Willard D. Hubbard

## Abstract

The general fluctuation theory for multicomponent systems has been applied in order to make corrections for the charge effect on systems of colloidal electrolytes where the gegenions and simple cations may have any valence. The basic equations of Prins and Hermans and Princen and Mysels were used for this derivation. The aggregation number and apparent charge were calculated from turbidity measurements on decyl-, dodecyl-, tetradecyl-, and hexadecyltrimethylammonium sulfate in different concentrations of Na_2_SO_4_, MgSO_4_, and La_2_(SO_4_)_3_ solutions and on dodecyltrimethylammonium bromide in the KBr, CaBr_2_, and LaBr_3_ solutions. The data indicate that the nature and concentration of the gegenion determine the size and charge of the micelle whereas the nature and concentration of the simple cation of the added electrolyte has little or no effect.

## 1. Introduction

Paraffin-chain salts are ionic compounds which have paraffin chains incorporated in one or both ions. Because of the hydrophobic nature of the paraffin-chain part there exists a threshold concentration, called the critical micelle concentration, *cmc*, above which the excess paraffin-chain ions associate to form aggregates of colloidal dimensions. The electrical charge of this aggregate is partially neutralized by an atmosphere of gegenions whose charge is of the opposite sign to the charge on the paraffin-chain ion. The resulting aggregate of paraffin-chain ions and gegenions is called a micelle.

Although micelles with univalent singly charged gegenions have been the subject of many physical chemical investigations [1–3]^1^ relatively little is known about micelles with gegenions of multiple charge, particularly in the presence of added electrolyte. The effect of the higher valence gegenions on the size, the *cmc*, and the charge on the micelle is important for a better understanding of the structure of these aggregates.

Light-scattering measurements provide an excellent means for obtaining information about these systems. Debye [4] was the first to provide a theory for determining the micellar weight by extrapolating the familiar $\frac{H\left(c-cmc\right)}{\tau }$ function to the *cmc*, the intercept being equal to 1/M^*^ where *c* is the concentration of the paraffin-chain salt, *H* the light-scattering constant, *τ* the excess turbidity, and M^*^ the micellar weight. Prins and Hermans [5] and Princen and Mysels [6], applying the general fluctuation theory for a multicomponent system, have made corrections for the charge effect on the micelles. The system they were concerned about was the micelle with an “apparent” charge, *p*, in the presence of the unmicellized ions and added 1:1 electrolyte having an ion in common with the paraffin-chain salt. They were able to obtain expressions for *p* and the aggregation number, *m*, in terms of the experimental quantities: the intercept *A* and the limiting slope *B* of the $\frac{H\left(c-cmc\right)}{\tau -\tau_{0}}$ versus (*c*−*cmc*) plot. They assumed that the apparent charge, *p*, absorbed all the non-idealities of the system.

It is the object of this paper to present general expressions for *p* and *m* with only the restriction that the added electrolyte has an ion in common with the paraffin-chain salt. This is the system of most interest in interpreting physical properties of the micelle.

The aggregation number and the apparent charge are calculated from turbidity measurements on decyl-, dodecyl-, tetradecyl-, and hexadecyltrimethylammonium sulfate in different concentrations of Na_2_SO_4_, MgSO_4_, and La_2_(SO_4_)_3_ solutions, and on dodecyltrimethylammonium bromide in KBr, CaBr_2_, and LaBr_3_ solutions.

## 2. Experimental Detail

### Materials

The alkyl bromides, obtained from the Fisher Scientific Company, were fractionally distilled through a Piras-Glover spinning band column, 60 cm in length. Only the center cuts having the same refractive index were used for the preparations. The *n*-alkyltrimethylammonium bromides were prepared by refluxing an excess (10%) of freshly distilled trimethylamine with the alkyl bromide in methyl alcohol for several hours. At the completion of the reaction the excess alcohol and trimethylamine were removed by distillation at room temperature under reduced pressure. The quaternary ammonium bromides were then recrystallized four or five times from different solvents.

The quaternary ammonium sulfates were prepared by reacting the corresponding bromides with an excess of silver sulfate in methyl alcohol. The insoluble silver salts were removed by filtration and the quaternary ammonium sulfates treated the same way as the bromides. The compounds were dried over P_2_O_5_ for one week before using.

Analyses for sulfate and bromide content agreed with the theoretical amount within experimental precision of 0.5 percent.

The lanthanum bromide was prepared by reacting lanthanum oxide in 48 percent HBr solution; the volatile matter was removed by vacuum distillation and the LaBr_3_ was recrystallized four times in methyl alcohol. Bromide analysis of this compound corresponded to LaBr_3_.7H_2_O.

The sodium sulfate, obtained from J. J. Baker Chemical Co., was dried at 110° for several hours and used without any further purification. The silver sulfate, obtained from Mallinckrodt, and the lanthanum sulfate, obtained from Fisher Scientific Co., were used without further purification.

### Apparatus and Procedure

All turbidity measurements were made at 436 m*μ* with a light-scattering photometer which has been previously described [9]. Refractive index increments at 436 m*μ* were determined in the usual manner with a differential refractometer similar to that used by P. P. Debye [10].

The dissymmetry (*I_x_*−*I_w_*)_45_/(*I_x_*−*I_w_*)_135_ was determined for all solutions. No values greater than 1.06 were obtained except for hexadecyltrimethylammonium sulfate in 0.01 *M* Na_2_SO_4_ solution which measured 1.10. No corrections were made for dissymmetry.

### Interpretation

The following derivations depend on five assumptions about the system: (1) the micelle contains *m* monomer ions with *m-p* gegenions bound to it. Its effective charge is *p<m.* (2) The concentration of simple inorganic ions is constant above the *cmc.* (3) The effective charge and size of the micelle is constant above the *cmc.* (4) The micelles are monodispersed. (5) The monomer and micelle may be treated as independent components. The validity of this model has been discussed in detail by Princen and Mysels [6] and by Prins and Hermans [5].

Interpretation of light-scattering data by multicomponent systems was first considered by Zernike [11] and more recently by Stockmayer [12] and Kirkwood and Goldberg [13]. Prins and Hermans [5] and Princen and Mysels [6] have applied it to paraffin-chain salt solutions. In the following derivation the basic equations analogous to those given by Prins and Hermans [5] will be used along with Mysels, method for extrapolating the $\frac{H\left(c-cmc\right)}{\tau -\tau_{0}}$ function to the *cmc*.

If a system contains *N*_1,_ *N*_2_….*N_s_* solute molecules of type 1, 2 …. *s* in a volume *V*, and the compressibility of the solution is not too different from that of the pure solvent, the excess turbidity of the solution over that of the solvent is

$$\tau =-\frac{RVD}{\Delta }$$ (1)

where $R=\frac{32\pi^{3}kTv^{2}}{3\lambda^{4}}$, *v* is the refractive index of the solution, λ the wavelength of the incident light in vacuo,

$$D=\left\|\begin{array}{cc} 0 & v_{j}\\ v_{i} & G_{ij} \end{array}\right\|;\Delta =\left|G_{ij}\right|i,\;j=1,2\ldots s$$

$$v_{i}=\frac{\left(\partial v\right)}{{\left(\partial N_{i}\right)}_{P,T}};G_{ij}=\frac{\left(\partial G_{i}\right)}{{\left(\partial N_{j}\right)}_{P,T}}=\frac{\left(\partial^{2}G\right)}{{\left(\partial N_{i}\partial N_{j}\right)}_{P,T}}$$

*G* is the Gibbs free energy and *G_i_* the partial derivative of *G* with respect to *N_i_.*

Consider a volume *V* containing *N*_0_ water molecules, *N*_1_ monomer ions with a valence of unity, *N*_3_ inorganic ions with valence Z_3_ of the same sign of charge as the monomer, and *N*_4_ micelles of charge *p* containing *m* monomers. Then under the requirement of electroneutrality, there will be

$$N_{2}=\frac{N_{1}}{Z_{2}}+\frac{Z_{3}N_{3}}{Z_{2}}+\frac{p}{Z_{2}}N_{4}$$ (2)

gegenions of valence *Z*_2_. The gegenion is common to both the paraffin-chain salt and the added electrolyte. Assuming ideal behavior, the Gibbs free energy is given by

$$\frac{G}{kT}=\sum_{j}N_{j}\left[\frac{u_{j}^{0}}{kT}+\ln\;X_{j}\right]\;j=0,1,2,3,4$$ (3)

where *X* is the mole fraction.

$$X_{j}=\frac{N_{j}}{S};\;S=\sum_{j}N_{j}=N_{0}+N_{1}\left(\frac{1}{Z_{2}}+1\right)+N_{3}\left(\frac{Z_{3}}{Z_{2}}+1\right)+N_{4}\left(1+\frac{p}{Z_{2}}\right).$$ (4)

The corresponding variables are *N*_1_, *N*_3_, and *N*_4_. The second derivatives of *G/kT* with respect to these variables are:

$$\begin{array}{l} G_{11}=\frac{1}{N_{1}}+\frac{1}{Z_{2}^{2}N_{2}}-\frac{1}{S}{\left(1+Z_{2}\right)}^{2}\\ G_{13}=\frac{Z_{3}}{Z_{2}^{2}N_{2}}-\frac{1}{S}\frac{Z_{2}Z_{3}+Z_{2}^{2}+Z_{3}+Z_{2}}{Z_{2}^{2}}\\ G_{14}=\frac{p}{Z_{2}^{2}N_{2}}-\frac{1}{S}\frac{Z_{2}^{2}+Z_{2}p+p+Z_{2}}{Z_{2}^{2}}\\ G_{33}=\frac{Z_{3}^{2}}{Z_{2}^{2}N_{2}}+\frac{1}{N_{3}}-\frac{1}{S}\left(\frac{Z_{3}^{2}+Z_{2}Z_{3}}{Z_{2}^{2}}+\frac{Z_{3}+Z_{2}}{Z_{2}}\right)\\ G_{34}=\frac{Z_{3}p}{Z_{2}^{2}N_{2}}-\frac{1}{S}\left[\frac{\left(Z_{2}+p\right)}{Z_{2}^{2}}+\frac{\left(Z_{2}+p\right)}{Z_{2}}\right]\\ G_{44}=\begin{array}{c} 1\\ N_{4} \end{array}+\frac{p^{2}}{Z_{2}^{2}N_{2}}-\frac{1}{S}(\frac{pZ_{2}+p^{2}}{Z_{2}^{2}}+\frac{Z_{2}+p}{Z_{2}}). \end{array}$$

At all concentrations except very high ones the last term in each of these expressions is small compared with the others and will therefore be omitted.

To a very good approximation we may substitute

$$v_{4}=mv_{1}$$ (5)

and introduce a term *f* defined by

$$f=\frac{v_{3}}{v_{1}}.$$ (6)

Substituting *D*, Δ, and *f* into eq (1) and rearranging terms:

$$\tau =RVv_{1}^{2}\frac{\left[\frac{N_{1}^{2}}{Z_{2}}+\frac{N_{1}N_{3}}{Z_{2}^{2}}\left(Z_{2}^{3}+Z_{2}Z_{3}+f^{2}Z_{2}+f^{2}-2fZ_{3}\right)+N_{3}^{2}Z_{3}f^{2}+\left(\Phi +\frac{M^{2}pN_{4}}{Z_{2}}\right)N_{4}\right]}{\frac{Z_{3}^{2}N_{3}}{Z_{2}^{2}}+\frac{Z_{3}N_{3}}{Z_{2}}+\frac{N_{1}}{Z_{2}^{2}}+\frac{N_{1}}{Z_{2}}+\frac{p}{Z_{2}}\left(1+\frac{p}{Z_{2}}\right)N_{4}}$$ (7)

where

$$\varphi =N_{1}\frac{p}{Z_{2}}+\frac{p^{2}}{Z_{2}^{2}}+\frac{m^{2}}{Z_{2}}+\frac{m^{2}}{Z_{2}^{2}}-\frac{2mp}{Z_{2}^{2}}+N_{3}\frac{m^{2}Z_{3}^{2}}{Z_{2}}+\frac{Z_{3}m^{2}}{Z_{2}}+\frac{f^{2}p}{Z_{2}}+\frac{f^{2}p^{2}}{Z_{3}^{2}}-\frac{2Z_{3}fmp}{Z_{2}^{2}}.$$

The experimental quantity of interest is *τ*—*τ*_0_ where *τ*_0_ is the value of *τ* when *N*_4_=0 (at the *cmc*).

$$\tau -\tau_{0}=\frac{RVv_{1}^{2}}{kT}\left[\frac{Y\Phi +\frac{Ym^{2}pN_{4}}{Z_{2}}}{Y^{2}+\frac{p}{Z^{2}}\left(1+\frac{p}{Z^{2}}\right)YN_{4}}\right]N_{4}$$ (8)

where

$$Y=\left(\frac{Z_{3}^{2}}{Z_{2}^{2}}+\frac{Z_{3}}{Z_{2}}\right)N_{3}+\left(\frac{1}{Z_{2}^{2}}+\frac{1}{Z_{2}}\right)\;N_{1}.$$

Let *C* be the concentration of paraffin-chain salt in g/ml, *C*_0_ the *cmc* in g/ml and *N_A_* Avogadro’s number.

$$N_{4}=\frac{N_{A}V\left(C-C_{0}\right)}{M_{4}}$$

where *M_4_* is the micellar weight. For *v*_4_ we substitute

$$\frac{vcM_{4}}{VN_{A}}\text{where}\;v_{c}=\left(\frac{\partial v}{\partial c}\right)P,T$$

This substitution is valid since in the range of concentration studied *V* may be considered a constant in the differentiation of *C.*

Finally the light-scattering constant *H* is equal to $\frac{Rvc^{2}}{kT}$ and eq (8) now becomes:

$$\frac{H\left(C-C_{0}\right)}{\tau -\tau_{0}}=\frac{g}{M_{4}}\left[\frac{Y^{2}+\frac{p}{Z_{2}}\left(1+\frac{p}{Z_{2}}\right)YN_{4}}{Y^{2}+g\frac{YpN_{4}}{Z_{2}}}\right]$$ (9)

where

$$\begin{array}{l} \;g=\frac{Y^{2}}{N_{1}^{2}d_{1}+N_{1}N_{3}d_{2}+N_{3}^{2}d_{3}}\\ d_{1}=\frac{p}{Z_{2}^{3}}+\frac{p}{Z_{2}^{4}}+\frac{m^{2}}{Z_{2}^{3}}+\frac{m^{2}}{Z_{2}^{4}}-\frac{2mp}{Z_{2}^{4}}+\frac{m^{2}}{Z_{2}^{2}}+\frac{m^{2}}{Z_{2}^{3}}-\frac{2mp}{Z_{2}^{3}}\\ d_{2}=\frac{2Z_{3}^{2}}{Z_{2}^{3}}+\frac{2Z_{3}^{2}}{Z_{2}^{4}}-\frac{2pZ_{3}^{2}}{Z_{2}^{4}m}+\frac{2Z_{3}}{Z_{2}^{2}}+\frac{2Z_{3}}{Z_{2}^{3}}-\frac{2Z_{3}p}{Z_{2}^{3}m}-\frac{2fZ_{3}p}{Z_{2}^{4}m}-\frac{2fZ_{3}^{2}p}{Z_{2}^{3}m}+\frac{2fZ_{3}p^{2}}{Z_{2}^{4}m^{2}}+\frac{2Z_{3}fp}{Z_{2}^{3}m^{2}}\\ d_{3}=\frac{Z_{3}^{4}}{Z_{2}^{4}}+\frac{2Z_{3}^{3}}{Z_{3}}+\frac{f^{2}Z_{3}^{2}p}{Z_{2}^{3}m^{2}}+\frac{f^{2}Z_{3}^{2}p}{Z_{2}^{4}m^{2}}-\frac{2fZ_{3}^{3}p^{2}}{Z_{2}^{4}m}+\frac{Z_{3}^{2}}{Z_{2}^{2}}-\frac{2Z_{3}^{2}fp}{Z_{2}^{3}m}. \end{array}$$

Expanding (9) in powers of concentration and substituting the value for *Y*

$$\frac{H\left(C-C_{0}\right)}{\tau -\tau_{0}}=\frac{gZ_{2}}{mM}\left[1+\frac{\left(p^{2}+Z_{2}p-pAMm\right)\left(C-C_{0}\right)}{\left(n_{1}\left[Z_{3}^{2}+Z_{2}Z_{3}\right]+n_{3}\left[1+Z_{2}\right]\right)\frac{Mm}{Z_{2}}}+0{\left(C-C_{0}\right)}^{2}\right]=A+B\left(C-C_{0}\right)$$ (10)

where *A* is the intercept and *B* is the limiting slope of the $\frac{H\left(C-C_{0}\right)}{\tau -\tau_{0}}-\left(C-C_{0}\right)$ line, *M* is the molecular weight of the paraffin-chain salt, and *n*_1_ and *n*_3_ are the concentration in moles/ml of the monomer and added electrolyte.

Rearranging (10) results in

$$m\left[\frac{BM\left\{\left(Z_{3}^{2}+Z_{3}Z_{2}\right)n_{3}+\left(1+Z_{2}\right)n_{1}\right\}+pAM}{AZ_{2}}\right]=p^{2}+Z_{2}p.$$ (11)

This gives an expression in m and *p* in terms of experimental quantities *B, A*, *n*_1_, and *n*_3_. For another expression in *m* and *p* consider the term *g*:

$$g=\frac{{\left[\frac{Z_{3}}{Z_{2}}\left(1+\frac{Z_{3}}{Z_{2}}\right)n_{3}+\frac{1}{Z_{2}}\left(1+\frac{1}{Z_{2}}\right)n_{1}\right]}^{2}}{n_{1}^{2}d_{1}+n_{1}n_{3}d_{2}+n_{3}^{2}d_{3}}=\frac{Am\;M}{Z_{2}}$$ (12)

rearranging terms

$$m^{2}+\frac{p^{2}F^{2}}{Z_{2}^{4}}+\frac{pF^{2}}{Z_{2}^{3}}-\frac{2pm\;F}{Z_{2}^{2}}=\frac{mZ_{2}}{AM}$$ (13)

where

$$F=\frac{n_{1}+f\;Z_{3}n_{3}}{\frac{Z_{3}^{2}}{Z_{2}^{2}}+\frac{Z_{3}}{Z_{2}}n_{3}+\frac{1}{Z_{2}^{2}}+\frac{1}{Z_{2}}n_{1}}.$$

Adding eqs (13) and (11)

$$m=\frac{Z_{2}}{AM}+\frac{2pF}{Z_{2}^{2}}-\frac{F^{2}BM\left[\left(Z_{3}^{2}+Z_{3}Z_{2}\right)n_{3}+\left(1+Z_{2}\right)n_{1}\right]}{Z_{2}^{5}A}-\frac{pAMF^{2}}{Z_{2}^{4}}.$$ (14)

This gives an expression for *m. p* may be determined by substituting the expression for *m* of eq (14) into eq (11) and solving the resulting quadratic equation

$$p=\frac{2BM\frac{\left[\left(Z_{3}^{2}+Z_{3}Z_{2}\right)n_{3}+\left(1+Z_{2}\right)n_{1}\right]}{Z_{2}^{3}}F+\sqrt{4B\left(n_{1}+n_{3}\right)\left(1+Z_{2}\right)}}{2A-\frac{2F\;A^{2}M}{Z_{2}^{2}}}.$$ (15)

Substituting *Z*_2_=*Z*_3_=l into eqs (14) and (15) gives Princen and Mysels [6] eqs (11) and (12) for the case where all the ions have a valency of unity.

## 3. Results and Discussion

**Figure 1:**
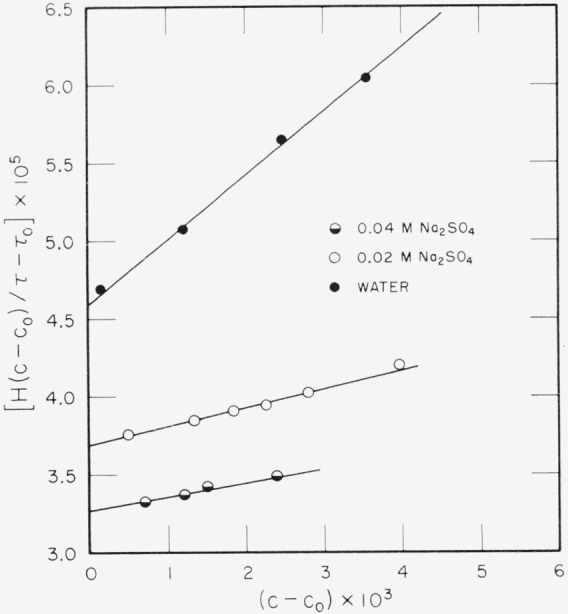
Light-scattering plots for tetradecyltrimethylammonium sulfate in water, 0.02 N, and 0.04 N Na_2_SO_4_ solutions.

In figure 1 are shown typical Debye $\frac{H\left(C-C_{0}\right)}{\tau -\tau_{0}}$ versus (*C*−*C*_0_) plots for tetradecyltrimethylammonium sulfate in water and in 0.02 *N* and 0.04 *N* Na_2_SO_4_ solutions. Values for the intercept, *A*, and the slope, *B*, for these and other measurements are given in table 1. Values for *f*, the ratio of the refractive index increment of added electrolyte to the refractive increments of the paraffin-chain salt, are also given in table 1.

Several authors [6, 7] have been concerned about the effect of the micellar charge on the determination of the aggregation number by means of turbidity measurements. They found for systems with univalent simple ions that the aggregation number obtained by Debye’s method [4] differs only slightly from those that were corrected for the charge effect. The same results were found in this investigation where the simple inorganic ions of the system had valences from one to three. In table 1 are given the values obtained by the Debye method, *m*, and those calculated from eqs (14) and (15).

Values for the apparent charge, *p*, calculated from eq (15) are given in table 1. These values for the sulfate micelles are lower than those obtained by Prins and Hermans [7] for the corresponding bromide micelles. Prins and Hermans found no clear-cut tendency of *p* to change with added salt content. The values of *p* obtained from this investigation appear to increase slightly with increasing salt content. However, it must be remarked that there is considerable experimental error in measuring the slope, *B*, of the $\frac{H\left(C-C_{0}\right)}{\tau -\tau_{0}}$ versus (*C*−*C*_0_) plots and therefore the calculation of *p* is crude. In addition, the critical micelle concentration, *C*_0_, must be known very accurately in determining *B* since in the lower concentration regions *C— C*_0_ is a small value. The values of *C*_0_ given in table 1 are average values obtained from conductivity, diffusion [3, 14], and turbidity measurements taken in this laboratory.

Although the values of *B* and hence *p* are subject to experimental error they have little effect on the determination of the aggregation numbers by eq (14).

The increase in micellar weight of the sulfate micelles with increasing added electrolyte concentration is similar to the increase in micellar weight of the corresponding chloride micelle [3]. The bromide micelles [7], on the other hand, increase in size much more rapidly. Apparently the nature and concentration of the gegenion determine the size and charge of the micelle whereas the concentration and nature of the simple cation have little or no effect. Turbidity measurements of tetradecyltrimethylammonium sulfate in 0.02 *N* Na_2_SO_4_, 0.02 *N* MgSO_4_, and 0.02 *N* La_2_(SO_4_) indicate the same value of *p* and *m* within experimental error. This was also found to be the case for dodecyltrimethylammonium bromide in 0.0125 *N* KBr, 0.0125 *N* CaBr_2_, and 0.0125 *N* LaBr_3_ solutions.

## 4. Summary

Turbidity measurements on parafin-chain salts in solutions of extraneous inorganic salts were interpreted using expressions derived from the general fluctuation theory for multicomponent systems. It was found that the aggregation numbers obtained by Debye’s method differ only slightly from those that were corrected for the charge of the micelle. The nature and concentration of the gegenion determine the size and charge of the micelle whereas the nature and concentration of the simple cation have little or no effect.

## Figures and Tables

**Table 1:** Turbidity and pertinent data

Paraffin-chain salt	Solvent	10^6^*n*_1_	10^6^*n*_3_	10^5^*A*	10^3^*B*	*f*	*m*	m^*^	*P*
									
C–10 sulfate	Water	50.3	0	14.5	3.29	0	28	28	4.8
C–12 sulfate	Water	9.3	0	8.10	0.48	0	45	45	1.1
C–12 sulfate	Na_2_SO_4_	7.2	10	7.97	0.57	0.29	46	45	2.6
C–12 sulfate	Na_2_SO_4_	6.5	20	7.29	0.55	0.29	50	49	2.8
C–12 sulfate	Na_2_SO_4_	5.9	40	6.62	0.51	0.29	55	54	4.8
C–14 sulfate	Water	1.5	0	4.62	6.24	0	71	71	1.3
C–14 sulfate	Na_2_SO_4_	0.82	10	3.95	2.13	0.25	84	83	4.6
C–14 sulfate	Na_2_SO_4_	0.77	20	3.82	1.08	0.25	87	86	6.8
C–14 sulfate	Na_2_SO_4_	0.66	40	3.28	0.83	0.25	101	100	10.3
C–14 sulfate	MgSO_4_	0.89	20	3.87	1.09	0.30	86	85	8.94
C–14 sulfate	La_2_ (SO_4_)_3_	0.87	6.7	3.82	1.44	0.39	87	86	10
C–16 sulfate	Na_2_SO_4_	0.12	10.0	2.39	1.32	0.23	127	126	7.2
C–10 bromide	Water	68.6	0	11.5	4.20	0	31	31	6.6
C–12 bromide	Water	14.8	0	5.87	6.52	0	55	55	7.5
C–12 bromide	KBr	11.6	12.5	4.86	3.90	0.24	71	67	9.2
C–12 bromide	CaBr_2_.2H_2_O	11.3	6.25	4.83	3.59	0.38	73	67	9.6
C–12 bromide	LaBr_3_.7H_2_O	11.7	4.17	4.79	3.55	0.44	74	68	11.5

## Footnotes

1 Figures in brackets indicate the literature references at the end of this paper.
